# A surgical reduction technique for posterior cruciate ligament avulsion fracture in total knee arthroplasty: a comparison study

**DOI:** 10.1186/s13018-020-01810-7

**Published:** 2020-07-31

**Authors:** Wei Lin, Jinghui Niu, Yike Dai, Huaxing Zhang, Jing Zhu, Fei Wang

**Affiliations:** 1 Third Hospital of Hebei Medical University, Shijiazhuang, Hebei People’s Republic of China; 2 Hebei General Hospital, Shijiazhuang, Hebei People’s Republic of China; 3 Department of Orthopedic Surgery, Third Hospital of Hebei Medical University, No. 139 Ziqiang Road, Shijiazhuang, 050051 Hebei People’s Republic of China

**Keywords:** Posterior cruciate ligament, Avulsion fracture, Cruciate-retaining, Total knee arthroplasty

## Abstract

**Background:**

Posterior cruciate ligament (PCL) avulsion fracture of the tibia is an uncommon but serious complication during primary cruciate-retaining total knee arthroplasty (TKA). The first objective of this report was to conduct a retrospective cohort study to investigate the incidence and potential risk factors of PCL avulsion fracture in primary cruciate-retaining TKA. The second objective was to assess the functional outcomes of the knee after reduction of PCL avulsion fracture.

**Methods:**

From January 2014 to January 2016, 56 patients who experienced PCL avulsion fracture of the tibia in primary cruciate-retaining TKA were included in the study group. Patients in this group underwent reduction of avulsion fracture. In this period, we selected 224 patients (control group) for comparison. Patients in this group also underwent the same TKA, but no PCL avulsion fracture occurred. The range of motion of the knee and Knee Society Scores were assessed. The Forgotten Joint Score was used to analyze the ability to forget the joint. Differences were considered statistically significant at *p* < 0.05.

**Results:**

In our series, the incidence of PCL avulsion fracture was 4.6%. There were no significant differences (*p* > 0.05) with regard to the preoperative or postoperative range of motion of the knee, final 4-year mean clinical score in the study and control groups 92.4 ± 2.7 and 93.6 ± 1.9, respectively, and mean functional scores of 85.1 ± 1.8 and 87.1 ± 1.2, respectively.

**Conclusions:**

The incidence of PCL avulsion fracture of the tibia is relatively high. Older age and female gender were the two risk factors of fracture in primary cruciate-retaining TKA. Reduction of PCL avulsion fracture with a high-strength line can achieve good stability and function of the knee.

## Background

Osteoarthritis is a chronic joint disease that affects more than 100 million people in the world [1]. The knee joint is the most frequently affected joint, and total knee arthroplasty (TKA) is an effective method for the treatment of end-stage knee osteoarthritis [2]. In a posterior cruciate ligament (PCL)-retaining TKA, PCL avulsion fracture of the tibia is an uncommon but serious complication. However, the outcomes of reinsertion and reduction of PCL are rarely reported.

Both PCL and anterior cruciate ligament contribute stability of the knee. The normal physiological PCL drives physiological knee and provides a part of proprioceptive feedback by decreasing paradoxical roll forward and allowing the femur to execute a part of controlled rollback during flexion [3–5]. A large number of different techniques have been used to protect the PCL from injury. Liabaud et al. [6] reported the use of the bone island technique to protect the PCL and to preserve as much PCL as possible. In cruciate-retaining TKA, careful evaluation of the gap balance and tension of PCL is mandatory to prevent postoperative knee stiffness and instability. When performing a trial reduction, if the flexion gap is too tight, a PCL avulsion fracture may occur accidentally. Usually, this fracture is an incomplete fracture, so it is not necessary to convert to a posterior-stabilized TKA. In this setting, whether to reduce the PCL avulsion fracture or not becomes a controversial issue. Kim et al. [7] reported the incidence of tibial-sided PCL avulsion fracture during the primary cruciate-retaining TKA, but they did not attempt to reduce the fracture. As a result, a non-healing fracture, instability of the knee, and a failed TKA are the major concerns. Currently, few surgeons reported the reduction of PCL avulsion fracture of the tibia in primary cruciate-retaining TKA.

The first objective of this report was to conduct a retrospective cohort study to investigate the incidence and potential risk factors of PCL avulsion fracture in primary cruciate-retaining TKA. The second objective was to assess the functional outcomes of the knee after the reduction of PCL avulsion fracture.

## Materials and methods

Institutional Review Board approval was obtained before the study commenced. From January 2014 to January 2016, 56 patients who experienced PCL avulsion fracture of the tibia in primary cruciate-retaining TKA were included in the study group. Patients in this group underwent reduction of avulsion fracture. In this period, a total of 1216 primary cruciate-retaining TKAs were performed. Matching at a 1:4 ratio with regard to age, gender, body mass index (BMI), and follow-up time, we selected 224 patients (control group) for comparison. Patients in this group also underwent the same TKA, but no PCL avulsion fracture occurred. Our eligibility criteria were (1) unilateral knee osteoarthritis, (2) a primary cruciate-retaining TKA, (3) a knee with flexion-contracture deformity < 15°, and (4) a varus deformity < 20°. Patients who had knee instability, valgus knee, or stiff knee were excluded. All operations were performed in our center by the same senior orthopedic surgeon (WF) using the same surgical techniques.

### Surgical technique

**Fig. 1:**
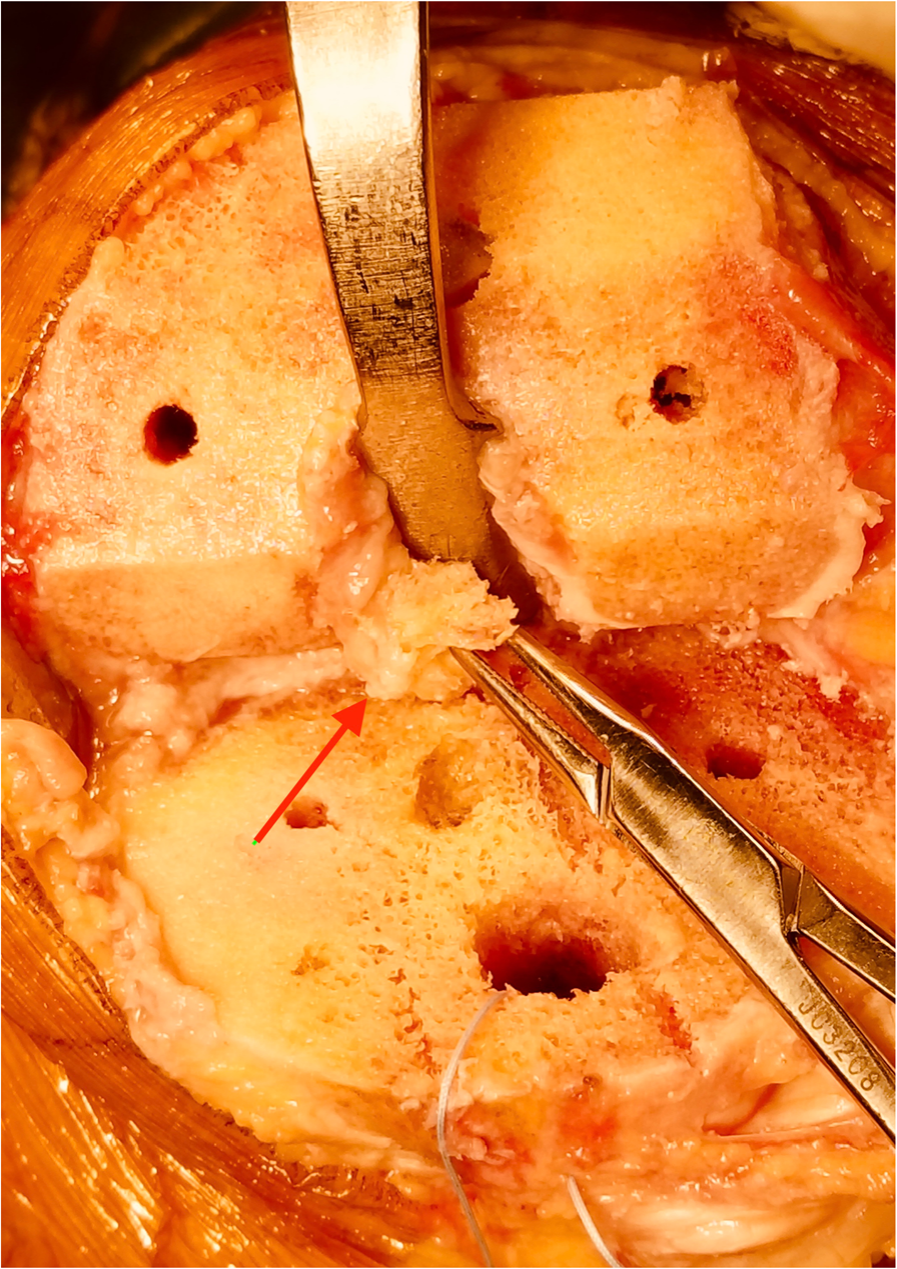
Intraoperative photo showing a PCL avulsion fracture (arrow)

**Fig. 2:**
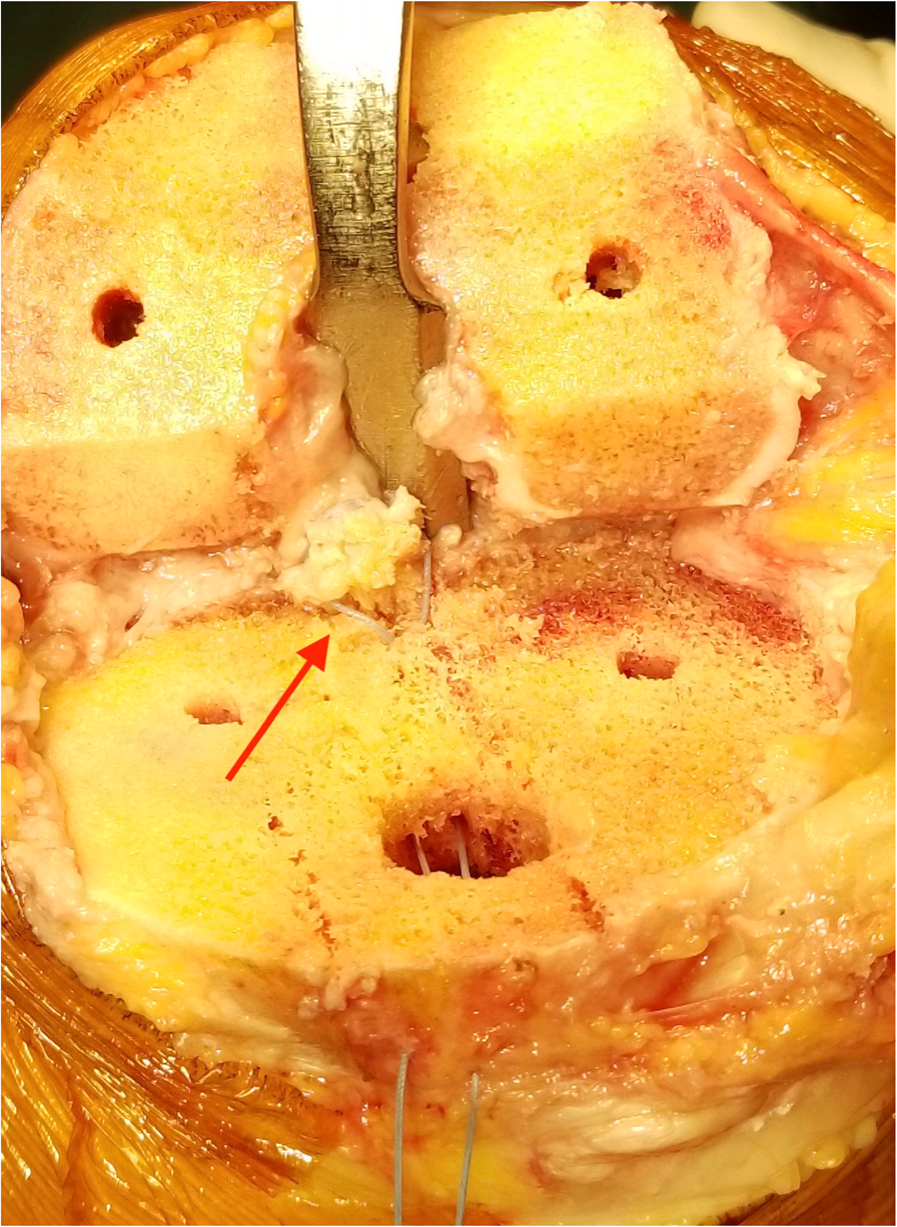
The PCL is sutured with a high-strength line (arrow)

**Fig. 3:**
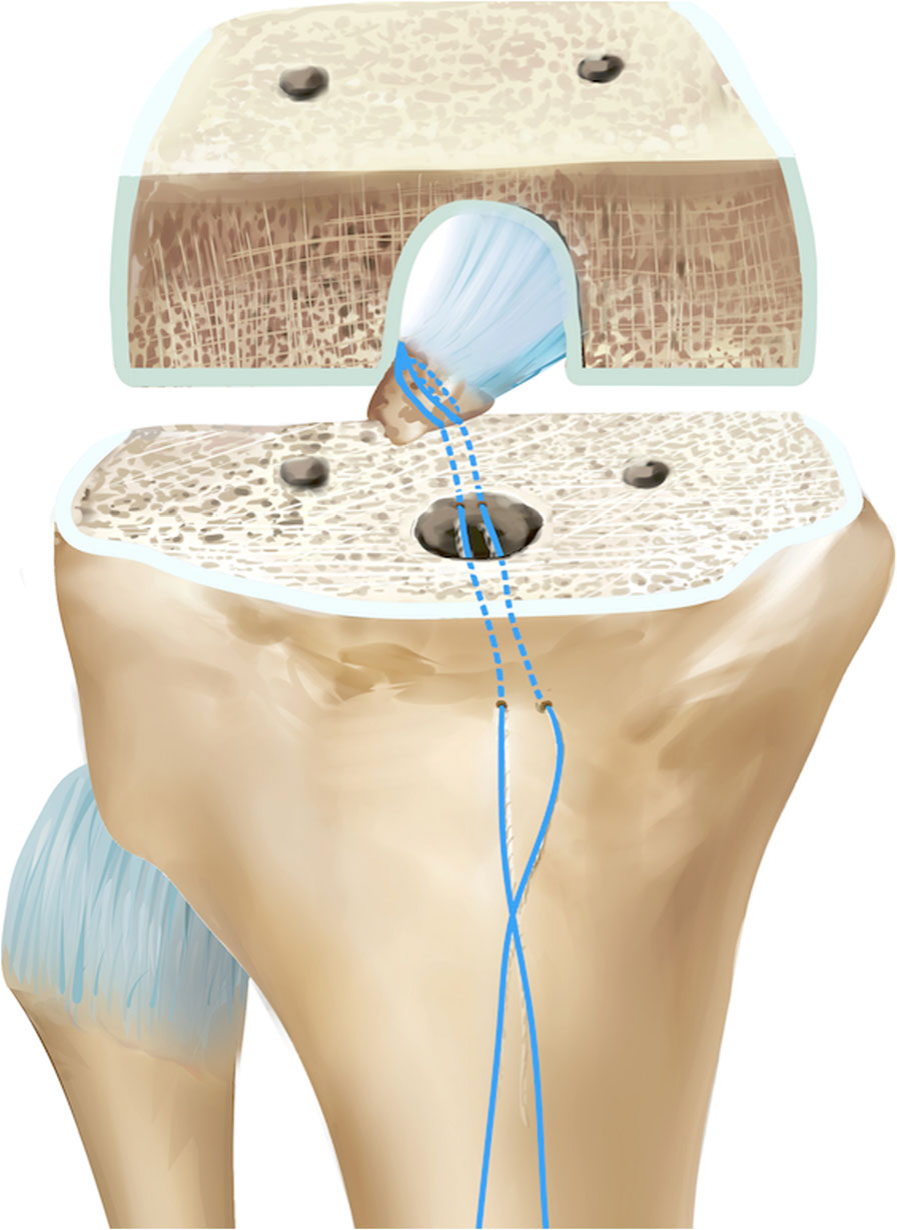
The PCL is sutured with a high-strength suture

In both groups, as described by Kim et al. [7], all primary cruciate-retaining TKAs were performed through the conventional midline skin incision. The distal femur cut was made with an intramedullary guide as the consultation to choose the right size of knee prosthesis. The anterior and posterior femoral condyles were resected with a battery-powered saw. For tibial resection, we made a bone island to protect the tibial attachment of PCL. Using an extramedullary cutting guide, a piece of 12-mm-thick bone was resected. The retroversion angle was 5 to 10°. Soft tissue balance was measured before trailing by using a tensor/balancer device as described by Sasanuma et al. [8]. During insertion and removal of trial components, if a PCL avulsion fracture of the tibia occurred accidentally (Fig. 1), reinsertion and reduction of PCL avulsion fracture were performed; this procedure was performed in the study group. A high-strength suture with needle was passed through the tibia plateau (Fig. 2). The distal insertion of PCL was sutured with the high-strength suture, and a high-strength suture knot was secured on the anterior cortex of the tibia (Fig. 3). The fracture was reduced, and the prosthesis was implanted to restore the gap balance. With the knee flexed, the high-strength suture was tightened and tied at the front of the tibia tubercle before bone cement dried. Thus, the high-strength suture was fixed with dried bone cement. All patients used the same knee prosthesis (LINK, Germany, Gemini MK II). After the total knee prosthesis was implanted, the wound was closed in layers.

### Postoperative managements

All patients received the same postoperative pain control and rehabilitation programs. Muscle exercises on the lower limbs and range of motion exercises ideally started 1 day after surgery [9]. All the patients were required to walk with crutches to support part of the weight. As soon as the patients regained adequate quadriceps control, crutches would be discontinued.

### Outcome evaluation

Assessments were performed by a senior orthopedic surgeon (DYK) who did not attend the treatments. The range of motion of the knee and Knee Society Scores (KSS; including clinical and functional scores of the knee) [10] were assessed. For comparing the postoperative status of the osteoarthritic patients after TKA, we used the Forgotten Joint Score (FJS; a 12-item questionnaire with a maximum of 100) to analyze the ability to forget the joint [11, 12]. Higher scores represented better results. Knee stability was assessed with the drawer test by the senior orthopedic surgeon (DYK). Fracture healing was defined as a callus bridging the fracture fragment on X-ray.

### Statistical analysis

In order to determine whether age, gender, body mass index, preoperative range of motion, and component size were the potential risk factors of PCL avulsion fracture, we used logistic regression to analyze the correlation. The normality of continuous variables was checked with Shapiro-Wilk’s test. If the data were normally distributed, the two groups were compared using the Student *t* test; on the contrary, a non-parametric test was selected. Categorical variables were checked with the chi-square test or Fisher’s exact test. Logistic regression was used to identify the potential risk factors of PCL avulsion fracture. The data were analyzed with SPSS 19.0 (SPSS, Chicago, IL, USA). Differences were considered statistically significant at *p* < 0.05.

## Results

**Fig. 4:**
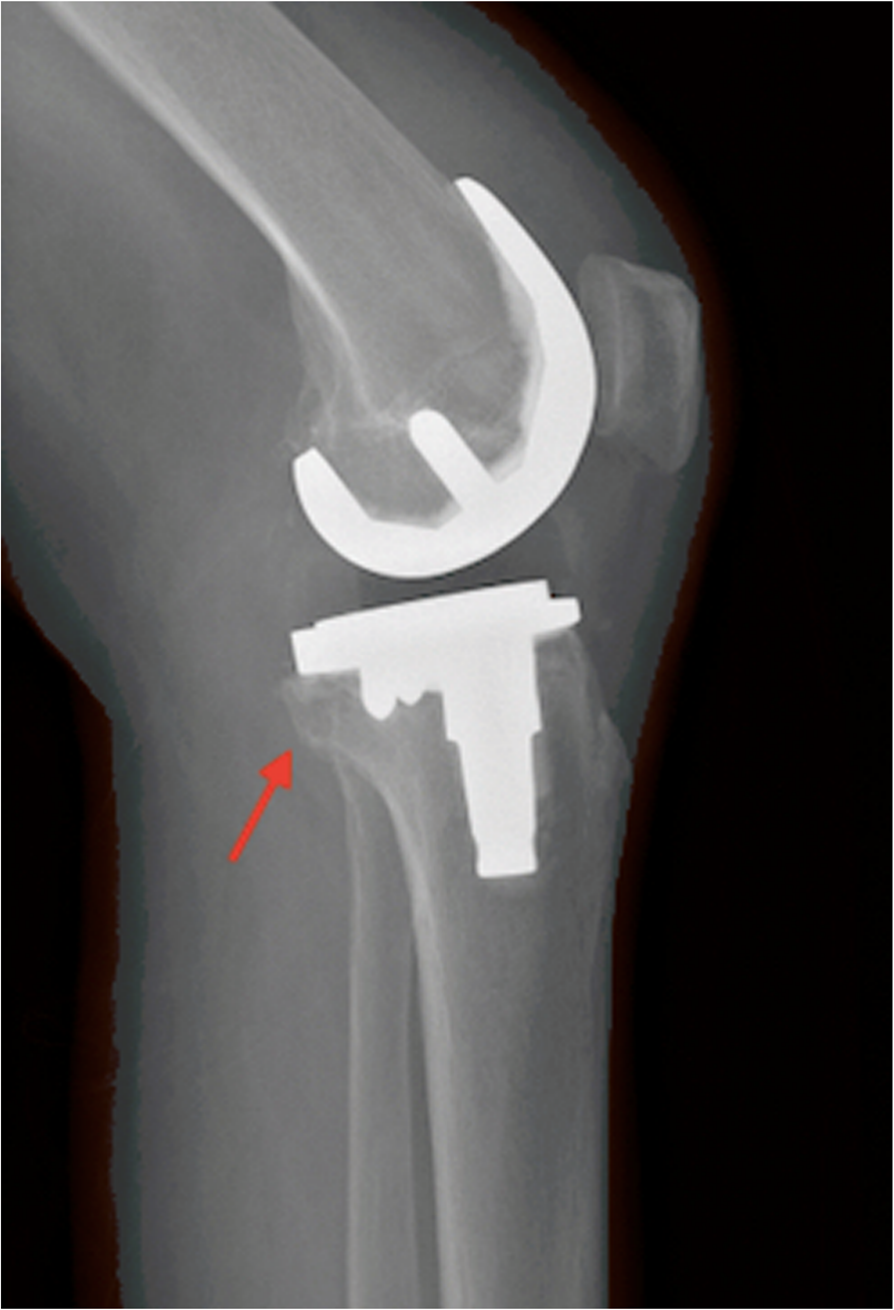
Lateral X-ray showing bone healing after 3 months

In the study group, complete reduction was achieved and maintained in all patients. Based on the radiographic evaluation, fracture healing occurred in all patients within 3 months (Fig. 4). No patient experienced knee instability. No patient walked with an assistive device, and no patient underwent a revision surgery.

Patient demographics and outcomes of both groups were summarized in Tables 1 and 2. In our series, the incidence of PCL avulsion fracture was 4.6% (56:1216) in primary cruciate-retaining TKA. We found no significant differences between the study and control groups with regard to age, gender, body mass index, size of components, or follow-up period. We found no significant difference with regard to preoperative or postoperative range of motion of the knee. At the final follow-up, the mean clinical scores of the study and control groups were 92.4 ± 2.7 and 93.6 ± 1.9, respectively (*p* = 0.248). The mean functional scores were 85.1 ± 1.8 and 87.1 ± 1.2, respectively (*p* = 0.066). We found no significant statistical difference in regard to FJS (*p* = 0.426). The results of logistic regression analysis demonstrated that older age and female gender were the two risk factors of PCL avulsion fracture (Table 3).

**Table 1:** Patient demographics for the study and control groups

Demographics	Study group	Control group	*p* value
Total patients	56	221	–
Age (years)	70.6 ± 4.2	66.3 ± 6.8	0.041
BMI (kg/m^2^)	27.3 ± 4.2	28.5 ± 3.6	0.183
Gender			0.048
Male	12 (21.4%)	78 (35.3%)	–
Female	44 (78.6%)	143 (64.7%)	–
Component sizes			
Femur	3 (1–6)	3 (1–6)	0.251
Tibia	3 (1–7)	4 (1–7)	0.362
Follow-up (years)	4.1 ± 0.3	4.2 ± 0.4	0.661

*BMI* body mass index, mean ± standard deviation

**Table 2:** Clinical and functional outcomes for the study and control groups

	Study group	Control group	*p* value
ROM			
Preop	96.2 ± 8.4	97.1 ± 8.7	0.651
Last follow-up	115.6 ± 7.2	117.9 ± 6.8	0.495
KSS			
Clinical score			
Preop	36.6 ± 4.2	37.1 ± 3.6	0.774
Last follow-up	92.4 ± 2.7	93.6 ± 1.9	0.248
Functional score			
Preop	38.1 ± 4.2	38.3 ± 3.1	0.819
Last follow-up	85.1 ± 1.8	87.1 ± 1.2	0.066
FJS			
Last follow-up	82.5 ± 3.4	83.1 ± 2.8	0.426

*KSS* Knee Society Score, *FJS* Forgotten Joint Score, *Preop* preoperation, mean ± standard deviation

**Table 3:** Logistic regression analysis of the risk factors

	Odds ratio	95% CI	*p* value
Age (years)	1.875	1.617–5.292	0.032
Gender	1.927	1.031–7.138	0.041
BMI (kg/m^2^)	0.724	0.732–1.031	0.731
Preoperative ROM	0.816	0.831–1.021	0.417
Component sizes	0.853	0.819–1.052	0.513

*BMI* body mass index, *ROM* range of motion, *CI* confidence interval

## Discussion

Our study found that the incidence of avulsion fracture in primary cruciate-retaining TKA was relatively high, and older age and female gender were the two risk factors. Our reinsertion and reduction technique was a reliable treatment for PCL avulsion fracture. By using the technique, acceptable PCL function was maintained, which provides sufficient stability to the knee during movement.

There are few studies that investigate patient-reported outcomes of PCL reduction in primary cruciate-retaining TKA. Kim et al. [7] showed that the incidence of the tibial-sided PCL avulsion fractures was 1.7%, and female gender was the only risk factor. However, they did not reduce the PCL avulsion fracture, and the function of PCL was assessed based on the comparison between the pre- and postoperative range of motion of the knee. In our comparison cohort study, we assessed the function of PCL based on both the objective scores (Knee Society Scores) and the subjective scores (FJS). Those multiple assessments enable the creation of a more comprehensive understanding of PCL function, ultimately leading to more accurate and appropriate clinical conclusions.

Our study showed that the incidence of the fracture was 4.6%, relatively higher, and we thought this may be related to a high (12 mm thickness) tibial osteotomy as required by the instructions for fitting the prosthesis. Therefore, we should be more cautious in the selection of the prosthesis in the future. In our experience, PCL avulsion fractures may occur when the flexion gap was too tight. In addition, older age and female gender are the two risk factors, because osteoporosis affects mostly older women. We believe that this technique is more applicable to incomplete PCL avulsion fracture, and as the repair has not been biomechanically tested, yet it is being used for a very important function to ensure PCL stability postoperatively. However, when the flexion gap was too tight or PCL reinsertion was difficult, we would convert to a posterior stabilized prosthesis.

Both the anterior cruciate ligament and PCL play important roles in maintaining optimal knee stability. In our study, resecting the anterior cruciate ligament may cause instability of the affected knee joint, but PCL has also been recognized as the limiting factor for the posterior translation of the tibia when the knee flexion is greater than 30° [6, 13]. Patients can tolerate the loss of PCL at rest, but this kinematic change often leads to severe knee dysfunction [14]. Some studies showed that preservation of PCL probably promoted patient proprioception, leading to increased patient satisfaction and knee feeling more “normal” after TKA [13, 15]. This finding corresponds to the FJS in our study. The FJS is a newly developed scoring system in recent years, which is often used to measure patients’ ability of forgetting joint replacement or joint awareness in daily life. In daily activities, people often do not realize their healthy joints, so we take the lack of awareness of normal healthy joints (forgotten joints) as the standard to assess the outcomes after TKA [11]. The FJS is affected by many factors, but we found that reduction of PCL avulsion fracture in primary TKA achieved acceptable FJS after 4 years. The PCL maintains the stability of the knee, contributes to good gap balance, and helps to maintain good proprioception [16]. Therefore, we believed that reduction of PCL avulsion fracture with high-strength suture could achieve good stability and function of the knee.

The proper tension of PCL is an important success factor in cruciate-retaining TKA [9]. Excessive release of the ligaments may result in worse outcomes [17–20]. In many cases, the PCL insertion may be damaged during the tibial cut, which raises a question about how much the PCL is actually preserved [17, 21–25]. Some surgeons argued that a posterior-stabilized TKA with a spine-cam mechanism is an alternative when the PCL is sacrificed. Moreover, whether the PCL should be preserved has been discussed for nearly 30 years. Either a cruciate-retaining TKA or a posterior-stabilized TKA has its advantages and disadvantages of process, clinical outcomes, kinematics, and lifespan [9, 26, 27]. In a human cadaveric study, Kennedy et al. [28] compared the kinematics of the knees after tibial resection with vs without preservation of the intact PCL, anterior lateral bundle, and posterior medial bundle. They found that the anterior lateral bundle and posterior medial bundle were the main stabilizers of the knee joint and serve primarily to resist the posterior translation of the tibia. Consequently, the surgeons should do their best to prevent PCL avulsion fractures in cruciate-retaining TKA.

Our research has several limitations. The retrospective study has a potential bias and weaknesses. The small number of PCL avulsion fracture decreases the power of the research. Surgeon preference, experience, and ability may influence ascertaining the effects of TKA. The follow-up period of 4 years is insufficient to fully assess the outcomes of PCL reduction.

## Conclusions

Our research showed that the incidence of PCL avulsion fracture of the tibia is 4.6%. Older age and female gender were the two risk factors in primary cruciate-retaining TKA. Reduction of PCL avulsion fracture with high-strength suture can achieve good stability and function of the knee.

## Data Availability

The detailed data and materials of this study were available from the corresponding author through emails on reasonable request.

## Abbreviations

PCL: Posterior cruciate ligament
TKA: Total knee arthroplasty
ROM: Range of motion
KSS: Knee Society Score
FJS: Forgotten Joint Score
BMI: Body mass index
